# Central Resistance to Thyroid Hormones in Morbidly Obese Subjects Is Reversed after Bariatric Surgery-Induced Weight Loss

**DOI:** 10.3390/jcm9020359

**Published:** 2020-01-28

**Authors:** Paula Juiz-Valiña, María Cordido, Elena Outeiriño-Blanco, Sonia Pértega, Bárbara María Varela-Rodríguez, María Jesús García-Brao, Enrique Mena, Lara Pena-Bello, Susana Sangiao-Alvarellos, Fernando Cordido

**Affiliations:** 1 Endocrine, Nutritional and Metabolic Diseases Group, Faculty of Health Sciences, University of A Coruña, 15006 A Coruña, Spain; Paula.Juiz.Valina@sergas.es (P.J.-V.); Maria.Cordido.Carro@sergas.es (M.C.); barbara.varela.rodriguez@gmail.com (B.M.V.-R.);; 2 Instituto de Investigación Biomedica (INIBIC), University Hospital A Coruña, 15006 A Coruña, Spain; 3 CICA (Centro de Investigaciones Científicas Avanzadas), As Carballeiras, s/n Campus de, San Vicente de Elviña, 15008 A Coruña, Spain; 4 Department of Endocrinology, University Hospital A Coruña, 15006 A Coruña, Spain; Elena.Outeirino.Blanco@sergas.es; 5 Clinical Epidemiology and Biostatistics Unit, University Hospital A Coruña, 15006 A Coruña, Spain; Sonia.Pertega.Diaz@sergas.es; 6 Department of Digestive and General Surgery, University Hospital A Coruña, 15006 A Coruña, SpainEnrique.Mena.del.Rio@sergas.es (E.M.)

**Keywords:** obesity, bariatric surgery, endocrine abnormalities, thyroid hormone resistance

## Abstract

Endocrine abnormalities are common in obesity, including altered thyroid function. The altered thyroid function of obesity may be due to a mild acquired resistance to the thyroid hormone. The aim of this study was to investigate the effect of weight loss after bariatric surgery (BS) on resistance to thyroid hormones in patients with extreme obesity compared with a control group. We performed an observational study evaluating patients with extreme obesity who underwent BS. We included 106 patients (83 women) and 38 controls (24 women). The primary endpoint was the thyrotroph thyroxine resistance index (TT4RI) and thyroid stimulating hormone (TSH) index (TSHRI). The parameters were studied before and after surgery. TSHRI and TT4RI were higher in the obese patients than in the control group. TT4RI and TSHI decreased significantly over time after surgery, with this decrease being associated with the excessive body mass index (BMI) loss and C-reactive protein (CRP). In extreme obesity, BS promotes a significant decrease in the increased TT4RI and TSHI. This decrease of TT4RI and TSHI is progressive over time after BS and significantly associated with excess BMI lost and CRP. Extreme obesity is characterized by a mild reversible central resistance to thyroid hormones.

## 1. Introduction 

Obesity has reached epidemic proportions. Since 1980, the prevalence of obesity has continuously increased in most countries. The disease burden related to high BMI has increased since 1990 [1]. The prevalence of obesity in the United States in 2013–2014 was 35% among men and 40.4% among women. The corresponding values for morbid obesity were 5.5% for men and 9.9% for women [2]. Recent analysis indicates that the prevalence in the United States of adult obesity and severe obesity will continue to increase [3]. Similar, although slightly lower, results are found in Europe [4] and elsewhere [5]. A moderate 5% weight loss improves metabolic function in multiple organs simultaneously, and progressive weight loss causes dose-dependent alterations in key adipose tissue biological pathways [6]. Among obese patients, bariatric surgery (BS) using gastric bypass, or sleeve gastrectomy, compared with non-surgical obesity management, was associated with a more marked improvement in comorbidities and lower all-cause mortality [7].

Excess body fat is associated with endocrine alterations, including decreased GH secretion [8,9,10] and thyroid dysfunction [10,11]. Thyroid hormone levels have been reported to be normal, increased, and decreased in obese patients; this discrepancy probably reflects the fact that the patients were examined at different times and may differ in degree and type of obesity and metabolic complications [11,12,13,14]. The alteration of thyroid function and the effect of BS on thyroid function is not fully understood. There are previous studies showing different results regarding the variation of thyroid stimulating hormone (TSH) after weight loss surgery [15,16,17,18,19]. The free thyroxine (FT4) values in obesity and the variation after surgery are even more conflicting [15].

Thyroid hormones (TH) play a central role in energy metabolism and body weight balance, and TH function correlates with energy expenditure and body weight [20]. Hypothyroidism is associated with hypometabolism, reduced lipolysis, and weight gain. On the contrary, hyperthyroidism is a hypermetabolic situation characterized by increased lipolysis and weight loss [20]. TH and TSH are inversely correlated due to negative feedback. Nevertheless, high thyroxine coexists with high TSH in patients with resistance to the thyroid hormone, an inherited autosomal recessive disorder [21,22]. Resistance to TH manifestations can be classified into central resistance, which affects the feedback set point in the pituitary, and peripheral resistance, which decrease the thyroid hormones’ metabolic effects. The central resistance can be evaluated by measuring thyroid hormones and TSH, or more accurately with indices derived from them [23,24]. In morbidly obese individuals there is a tendency to have higher levels of both thyroxine and TSH [25]. Therefore, given the thyroid feedback loop, the previously mentioned reports showing an association between high TH or high TSH and obesity provide seemingly contradictory results. These conflicting results may be conciliated if high thyroxine and high TSH occurrence in obesity reflected a central resistance to thyroid hormones. This central resistance would probably be the expression of a generally reduced sensitivity to thyroid hormones that is also peripheral. An acquired mild resistance to TH, due to homeostatic compensation, has been hypothesized [26]. Evidence of the association between indices measuring resistance to TH and prevalence of obesity, diabetes, and metabolic syndrome has recently been found [27].

This alteration of hypothalamus–pituitary axis sensitivity to thyroid hormones has not been studied before and after weight loss. In this work, we hypothesized that morbid obesity is a clinical situation of acquired resistance to thyroid hormones, probably due to homeostatic compensation, which is reversible with weight loss. The aim of this study was to investigate the effect of weight loss after bariatric surgery on indices of central resistance to thyroid hormones in clinically euthyroid patients with morbid obesity.

## 2. Patients and Methods

### 2.1. Patients and Controls 

We performed a retrospective observational study evaluating patients with extreme obesity and normal preoperative thyroid function that underwent BS in the University Hospital of A Coruña between January 2016 and December 2018. Inclusion criteria were BMI above 40 Kg/m^2^ or BMI above 35 Kg/m^2^ with at least one obesity-related comorbidity. Exclusion criteria were: history of thyroid disease, treatment with thyroid hormone, antithyroid drugs, amiodarone, or lithium, and secondary obesity. The study protocol was approved by our center’s ethics committee (Xunta de Galicia, approval code number: 2014/135), and written informed consent was obtained from all patients and controls. All of the studies were conducted in accordance with the Declaration of Helsinki. We included a total of 144 patients and controls (107 women) in our study, of which there were 106 patients (83 women) and 38 controls (24 women) selected from a pool of volunteers available to our unit. None of the controls had diabetes or other medical problems, nor were they taking any type of medication.

### 2.2. Parameters Evaluated

The following parameters were evaluated: age, sex, body mass index (BMI), body fat percentage, excessive BMI loss in percentage (EBMIL), TSH, free T4 (FT4), thyrotroph thyroxine (T4) resistance index (TT4RI), TSH index (TSHRI), insulin, HOMA-IR, C-reactive protein (CRP), GH, IGF-1, C peptide, and the type of BS performed (Roux-en-Y gastric bypass (RYGB) or sleeve gastrectomy (SG)). The parameters were evaluated before and 1, 3, 6, and 12 months after surgery. All blood samples were collected and immediately centrifuged, separated and frozen at −80 °C. Total body fat was calculated through bioelectrical impedance analysis (BIA). The primary end point was TT4RI and TSHRI.

### 2.3. Assays and Other Methods

Serum TSH (mIU/L) was measured by a two-site chemiluminescent immunoassay (ADVIA Centaur, Siemens, Deerfield, IL, USA) with a sensitivity of 0.01 mIU/L and with intra-assay coefficients of variation of 2.5%, 2.4%, and 2.4% for low, medium, and high serum TSH levels, respectively; and with inter-assay coefficients of variation of 5.3%, 3.4%, and 2.1% for low, medium, and high TSH levels, respectively; as previously published [19]. Serum FT4 (ng/dL) was measured by a direct chemiluminescent immunoassay (ADVIA Centaur, Siemens) with a sensitivity of 0.1 ng/dL and with intra-assay coefficients of variation of 3.3%, 2.2%, and 2.5% for low, medium, and high plasma FT4 levels, respectively; and with inter-assay coefficients of variation of 2.5%, 4.0%, and 2.3% for low, medium, and high FT4 levels, respectively; as previously published [19]. Insulin (µU/mL) was determined with a chemiluminescent immunometric assay (Immulite 2000 Insulin, DPC, Los Angeles, CA, USA) and with an intra-assay CV of 5.5%, 3.3%, and 3.7% for low, medium, and high plasma insulin levels, respectively; and with an inter-assay CV of 7.3%, 4.1%, and 5.3% for low, medium, and high insulin levels, respectively. Serum GH (µg/L) and IGF-1 (ng/mL) were determined by a chemiluminescent assay (Immulite, EURO/DPC) as previously published [19]. Plasma glucose (mg/dL) was measured with an automatic glucose oxidase method (Roche Diagnostics, Mannheim, Germany). All samples from a given subject were analyzed in the same assay run.

### 2.4. Calculations

EBMIL was calculated using the formula: ((preoperative BMI−current BMI) / (preoperative BMI−25)) × 100 

Insulin sensitivity (IS) was measured with HOMA-IR with the formula: fasting serum insulin (µU/mL) × fasting plasma glucose (mmol/L) / 22.5.

Thyrotroph T4 Resistance Index (TT4RI) was calculated as FT4 (pmol/L) × TSH (mIU/L) [23]. TSH index (TSHRI) was calculated as ln TSH (mIU/L) + 0.1345 × FT4 (pmol/L) [24].

### 2.5. Statistical Analysis 

Descriptive analysis was used in order to determine the characteristics of both the patients and the control group. Continuous data are expressed as mean ± standard error (SE) and/or median and interquartile range (IR). Non-numerical variables are expressed as frequencies and percentages.

The control subjects and obese patients were compared using the Mann–Whitney test for quantitative parameters and the Chi-squared and Fisher’s exact tests for qualitative parameters. The Wilcoxon signed-rank test was used to compare the preoperative and post-surgical values in the obese patients. Data Availability: All of the data analyzed during this study are included in this manuscript as well as in previously published articles cited in the references. If any information is required, please contact the corresponding author.

To assess the overall association of TSHRI and FT4RI with anthropometric, biochemical, and hormonal data during the post-surgery period, repeated measures correlation was calculated [28].

Generalized estimating equations (GEE) models, with the autoregressive correlation structure, were used to evaluate the trajectory of postoperative TSHRI and FT4RI, as well as to determine factors associated with their changes after BS. Bivariate GEE models were used in order to observe the effect of each variable on the change in TSHRI or FT4RI, taking time into account. Only those features significantly associated with TSHRI/FT4RI values in the bivariate analysis were finally included in a multivariate GEE model.

In the multivariate analysis, statistical analyses were performed with SPSS, version 24.0, and R, version 3.5.1., with the packages geepack and rmcorr added. *p*-values < 0.05 were considered as statistically significant. 

## 3. Results

### 3.1. Preoperative Characteristic of the Obese Patients and the Control Group

Preoperative characteristic of the obese patients and the control group are presented in Table 1. Among the 106 patients studied, 83 were women and the mean age was 47.5 ± 0.9 years (Table 1). The surgical procedures performed were RYGB and SG. Among the 38 controls studied, 24 were women and the mean age was 45.0 ± 1.5 years (Table 1). The two groups had similar sex and age as designed by the matching criteria. 

### 3.2. Fasting Serum Levels

Fasting glucose, lipids, CRP, and hormones (mean ± SE, Median (interquartile ranges)) are presented in Table 2. TSHRI levels were higher in the obese group than in controls; 3.5 ± 0.1 vs. 2.5 ± 0.1 for the obese and control groups, respectively. TT4RI levels were higher in the obese group than in the controls; 66.0 ± 5.5 vs. 28.9 ± 2.2 for the obese and control groups, respectively. Fasting C-reactive protein levels were higher in the obese group than in the healthy controls; 0.9 ± 0.1 vs. 0.2 ± 0.1 for the obese and control groups, respectively.

### 3.3. Evolution over Time of the Clinical and Analytical Parameters

The mean EBMIL as a percentage 12 months after bariatric surgery was 71.6 ± 2.4%. The biochemical and hormonal parameters in obese patients before and 12 months after BS are presented in Table 3. TSHRI levels significantly decreased in the obese patients after BS weight loss; 3.5 ± 0.1 vs. 2.5 ± 0.1 for the obese patients before and 12 months after surgery, respectively. TT4RI levels significantly decreased in the obese patients after BS weight loss; 66.0 ± 5.5 vs. 30.9 ± 2.3 for the obese patients before and 12 months after surgery, respectively.

**Figure 1:**
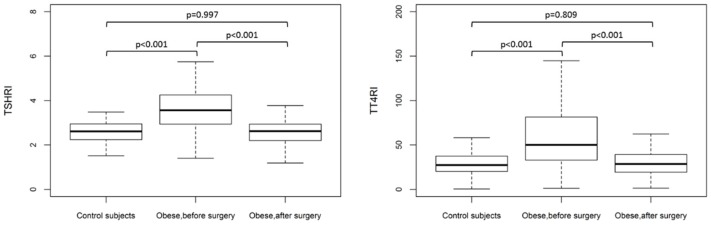
Thyrotroph T4 resistance index (TT4RI) and TSH index (TSHRI) values (Median (IR)) in control subjects (*n* = 38) and obese patients (*n* = 106) before and 12 months after surgery.

Figure 1 shows the TT4RI and TSHRI values (Median (IR)) in the control subjects and obese patients before and 12 months after surgery. TT4RI and TSHRI levels were higher in the obese group than in the healthy controls. TT4RI and TSHRI levels significantly decreased in the obese patients 12 months after surgery-induced weight loss.

**Figure 2:**
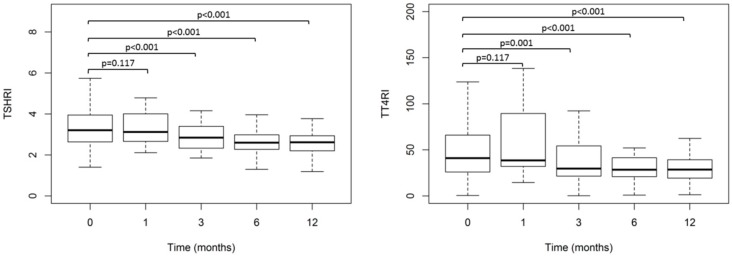
Evolution over time of Thyrotroph T4 resistance index (TT4RI) and TSH index (TSHRI) values (Median (IR)) before and after surgery (0, 1, 3, 6, and 12 months) in obese patients (*n* = 106).

Figure 2 shows the evolution over time of TT4RI and TSHRI (Median (IR)) before and after surgery (0, 1, 3, 6, and 12 months) in obese patients. The results show a decrease of TT4RI and TSHRI. The indices of central resistance to TH (TT4RI and TSHRI) significantly decreased in the obese patients at 3, 6, or 12 months after BS, when compared with presurgical values. TT4RI and TSHRI values were similar when compared with 3, 6, and 12 months after BS. 

**Figure 3:**
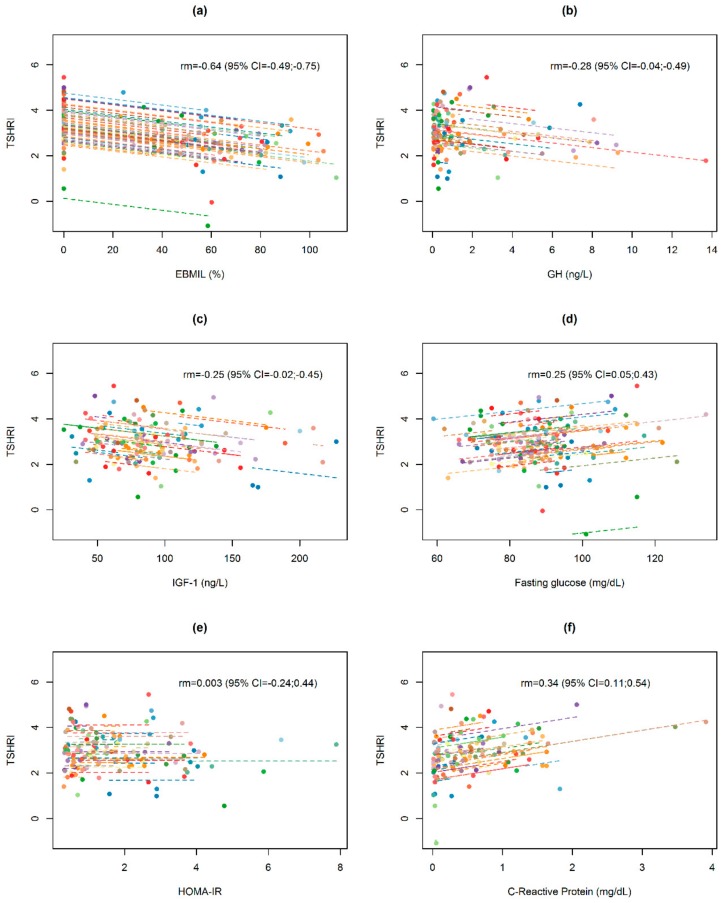
Repeated measures correlation for the overall relationship between changes in TSH resistance index (TSHRI) and (**a**) excessive BMI loss in percentage (EBMIL), (**b**) GH, (**c**) IGF-1, (**d**) Fasting glucose, (**e**) HOMA-IR and (**f**) C-reactive protein. Obese subjects (*n* = 106) are represented by dots than correspond to basal and postoperative TSHRI values and the respective anthropometric, biochemical, and hormonal data. Each line represents the repeated measures correlation fit for each participant.

**Figure 4:**
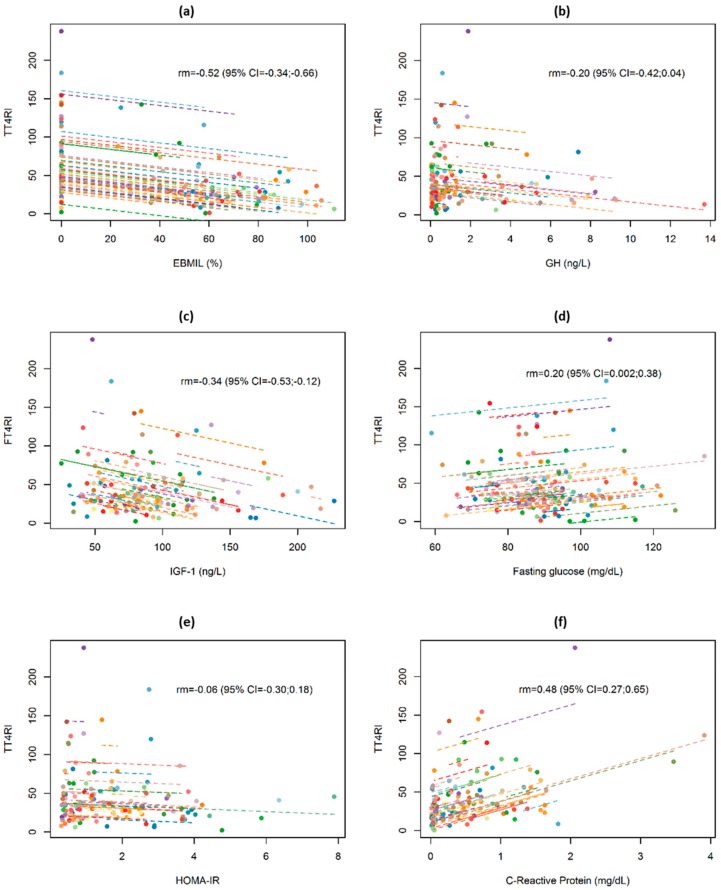
Repeated measures correlation for the overall relationship between changes in thyrotroph T4 resistance index (TT4RI) and (**a**) excessive BMI loss in percentage (EBMIL), (**b**) GH, (**c**) IGF-1, (**d**) Fasting glucose, (**e**) HOMA-IR, and (**f**) C-reactive protein. Obese subjects (*n* = 106) are represented by dots that correspond to basal and postoperative TT4RI values and the respective anthropometric, biochemical, and hormonal data. Each line represents the repeated measures correlation fit for each participant.

Post-surgery changes in TSHRI and TT4RI values in relation to changes in selected anthropometric, biochemical, and hormonal data are presented in Figure 3 and Figure 4. Changes in both indices were significantly negatively correlated with EBMIL (within-subjects repeated measures correlation rm = −0.64 and rm = −0.52, respectively) and significantly positively correlated with changes in CRP values (rm = 0.34 and rm = 0.48, respectively), but not correlated with changes in GH, IGF-1, fasting glucose, and HOMA-IR. Similar results were obtained from bivariate GEE models. 

After adjusting for EBMIL and CRP in a multivariate model, both variables were significantly and independently associated with TSHRI and TT4RI values in the follow-up, with a higher excess weight loss associated with lower thyroid resistance indices, whilst higher C-reactive protein values were associated with higher values in both indices (Table 4).

## 4. Discussion

The main result of this study is that we have found that the indices of central resistance to TH are increased in patients with extreme obesity, and that weight loss after BS leads to a decrease in these indices through direct or indirect mechanisms. The decrease in the indices of central resistance to thyroid hormones is significantly and independently associated with excess BMI lost and CRP. The present study strongly suggests that obesity is a situation of mildly increased pituitary resistance to TH that is reversed with BS. As far as we are aware, this is the first time that indices of resistance to thyroid hormone have been evaluated in morbidly obese subjects before and after lifestyle intervention, pharmacotherapy, or surgery-induced weight loss.

Different circulating TH levels have been found in obesity [12], although the majority of the studies have only been carried out on TSH levels. In agreement with our results, most studies have found increased TSH levels in patients with morbid obesity. Rotondi et al. [13] reported increased TSH levels in morbidly obese patients. Reinehr et al. [14] have found elevated TSH levels in obese children. Increased FT4 in obesity has been found in some studies [14]. Several studies have reported different results regarding the variation of TSH after BS and the relation of TSH decrease with weight loss [15,16,17,18]. Most [15,17], but not all [18] studies have found a decrease in circulating TSH after the operation. In agreement with our results, Guan et al. [15] found that bariatric surgery was associated with a decrease in circulating TSH levels. Neves et al. [17] found that BS promotes a significant decrease of circulating TSH. In obese children, weight loss was associated with a decrease in TSH, thyroid volume, and structure, while FT4 remained unchanged. These data suggest that the alterations of thyroid function and structure in patients with obesity are common and reversible [29]. Elsewhere, Dall’Asta et al. [18] evaluated obese subjects with normal thyroid function before and after weight loss through BS and found that TSH levels did not change. In the meta-analysis of Guan et al., although bariatric surgery was associated with a significant decrease in TSH, FT4 was not significantly changed postoperatively [15]. In accordance with these data, a recent study has found that TSH levels decreased in parallel with decreased BMI after bariatric surgery. However, no significant change was observed in FT4 or FT3 levels [30]. The differences between studies may be due to the type of bariatric surgery performed, the characteristics of the control group and the studied patients, or to the statistical power of the studies. As far as we are aware the indices of central resistance to thyroid hormones have not been studied before and after bariatric surgery.

The mechanism and the clinical implications of TSH and FT4 elevation in obese patients is still not fully understood. Our results are in agreement with the findings of Laclaustra et al. that found evidence of the association between indices measuring resistance to TH and the prevalence of obesity and diabetes in the US population [27]. TH signaling is unique for each cell (tissue or organ), depending on circulating thyroid hormone levels and on the exclusive blend of membrane transporters, deiodinases, and thyroid receptors present in each cell [31]. The resistance to thyroid hormone indices measures central sensitivity and the degree of pituitary gland inhibition by thyroxine. Peripheral resistance could also be present because, despite having a higher thyroxine, the studied group has morbid obesity, a phenotype that is physiopathologically associated with hypothyroidism [27]. Moreover, there are several animal models of reduced TH signaling in the presence of obesity [31,32]. TH receptors are less expressed on adipocytes of obese vs. lean individuals [33]. In addition, the TH receptor β has been found to be inversely correlated with disease severity in liver biopsies from patients that underwent bariatric surgery with different stages of obesity-induced nonalcoholic steatohepatitis [34]. Moreover, reduced sensitivity to TH could be epigenetically modified [35]. The reduced TH receptor expression may induce a decrease in hormone action, thereby increasing plasma TSH and constituting a condition of peripheral thyroid hormone resistance [33]. In the present study, we found increased indices of central resistance to TH in obese subjects which decreased after weight loss in accordance with the hypothesis that the increase of TSH and FT4 may represent a compensatory activation of the thyroid axis [12]. 

The mechanisms underlying the reason why the indices of central resistance to TH decrease after bariatric surgery remain unknown. We have found that the decrease in the indices of central resistance to TH is significantly associated with excess BMI lost after BS. This decrease is not due to an intrinsic effect of BS. A decrease in TSH has been found in obese patients after non-surgical induced weight loss [36] and the decrease in circulating TSH has been found to be associated with excess body weight loss after BS [17,19]. The present results show a decrease in the indices of central resistance to TH that is statistically significant 3 months after BS, and there was no further significant decrement, maybe due in part to the faster excess BMI loss in the first months after BS. In agreement with these data, the reduced TH receptor is reversed by weight loss, improving the thyroid hormone sensitivity [33]. One of the plausible explanations is the decrease of leptin levels following bariatric surgery [37]. With a decreasing amount of fat, the decreasing leptin [37] could promote a decrease of circulating TSH levels. However, leptin treatment was not associated with changes in thyroid size or nodularity or function [38]. Another potential mechanism may be insulin resistance: patients with extreme insulin resistance have a high prevalence of thyroid nodules, and patients with a homozygous insulin receptor mutation have significantly enlarged thyroid glands [38]. In the present study we could not find a significant influence of the insulin resistance indices on the central resistance to TH indices. Another potential mechanism may be HbA1c [39], however in the present article we could not find a significant influence of the HbA1c value on the resistance to TH indices. There is an important relationship between the GH-IGF-1 axis and obesity [8,9,10,40], and circulating GH modulates the thyroid axis. Circulating GH levels increased serum FT3 and decreased serum FT4 in humans [41]. Treatment with GH in adults with GH deficiency causes variable changes in thyroid function, the most consistent effect being decreased circulating thyroxine levels [42]. GH replacement therapy in a large group of adult GH-deficient patients has been shown to induce a significant reduction in FT4 [43]. The decreased GH secretion of obesity could be a contributory factor to the increased circulating FT4 levels. We explored the correlation between the GH-IGF-1 axis and the indices of central resistance to thyroid hormones, and were unable to find any important correlation. By contrast, we found that the variation of the increased indices of inflammation such as CRP highly correlate with the thyroid hormone resistance index in obese subjects. In agreement with these data, laparoscopic sleeve gastrectomy promotes TSH reduction in patients with morbid obesity, which correlates with an improved inflammatory state after surgery [44]. In any event, our results show that the decrease of the indices of central resistance to TH are associated with body weight loss after BS and CRP, suggesting that the decrease in the indices of central resistance to TH is mainly weight-mediated and probably due in part to the improved inflammatory state after surgery. Our results also suggest that the modification of thyroid function could be a potential therapeutic aim in patients undergoing BS [45].

We must acknowledge a number of limitations of our study. First, the relatively small sample size of our control subjects or obese patients, which did not allow for the stratification of different subgroups in the analysis. Secondly, we did not consider certain variables that could influence the study, such as the concomitant use of other medications. Thirdly, the heterogeneous nature of our patient group, with different obesity-associated comorbidities, secondary hormonal changes, and treatments, which could interfere with the thyroid hormone axis. However, there are several strengths to our study. We included sex- and age-matched controls in order to reduce the chances of misclassifying individuals due to variability in these variables. We evaluated TT4RI and TSHRI at different time points after BS, as most studies evaluated the variation of TSH alone using only two moments (before and after surgery). Moreover, this is the first study to evaluate the indices of central resistance to TH before and after BS. Nevertheless, more studies are required in order to better understand the complex relation between thyroid dysfunction and obesity [46].

## 5. Conclusions

In conclusion, this study shows that in patients with extreme obesity, weight loss induced with BS through direct or indirect mechanisms brings about a decline in the increased indices of central resistance to thyroid hormones. This decrease in central resistance to thyroid hormones is progressive over time after BS, and significantly and independently associated with BMI loss and CRP. Extreme obesity is characterized by a mild reversible central resistance to thyroid hormones.

## Figures and Tables

**Table 1:** Preoperative characteristic of the obese patients and the control subjects, (Mean ± SE; median, interquartile ranges).

	Control Subjects (*n* = 38)		Obese Subjects (*n* = 106)		
	Mean ± SE	Median (IR)	Mean ± SE	Median (IR)	*p*
Age (years)	45.0 ± 1.5	42.0 (37.3;53.0)	47.5 ± 0.9	46.9 (40.8;55.9)	0.149
Sex (*n*, %)					0.067
Female	24	63.2%	83	78.3%	
Male	14	36.8%	23	21.7%	
BMI (Kg/m^2^)	24.2 ± 0.6	23.7 (22.2;25.5)	49.7 ± 0.9	47.6 (43.6;53.3)	<0.001
Body fat (%)	26.5 ± 1.3	24.9 (20.5;32.2)	49.8 ± 0.7	50.8 (46.9;54.7)	<0.001
Diabetes (%)	0	0%	40	37.7	<0.001
HTA (%)	0	0%	52	49.1	<0.001
DL (%)	0	0%	35	33.0	<0.001
Type of surgery (%)					
Roux-en-Y gastric bypass			53	50.0	
Sleeve gastrectomy			53	50.0	

BMI, body mass index; HTA, hypertension; DL, dyslipidemia; IR, interquartile range.

**Table 2:** Biochemical and Hormonal data in control subjects and obese patients (Mean ± SE; median, interquartile ranges).

	Control Subjects (*n* = 38)		Obese Subjects (*n* = 106)		
	Mean ± SE	Median (IR)	Mean ± SE	Median (IR)	*p*
TSH (µU/mL)	2.0 ± 0.2	1.9 (1.3;2.4)	3.5 ± 0.3	2.7 (1.6;4.3)	0.001
Free T4 (ng/dL)	1.2 ± 0.1	1.1 (1.1;1.2)	1.5 ± 0.1	1.4 (1.3;1.6)	<0.001
TSHRI	2.5 ± 0.1	2.6 (2.2;2.9)	3.5 ± 0.1	3.6 (2.9;4.3)	<0.001
TT4RI	28.9 ± 2.2	27.4 (20.3;37.5)	66.0 ± 5.5	50.2 (33.0;81.4)	<0.001
Fasting Glucose (mg/dL)	88.9 ± 1.3	87.5 (84.0;93.0)	102.4 ± 2.8	97.0 (83.0;117.0)	0.014
HbA1c (%)	5.2 ± 0.1	5.2 (5.0;5.3)	7.2 ± 1.5	5.6 (5.2;6.0)	0.001
Fasting Insulin (µIU/mL)	5.2 ± 0.7	3.5 (2.8;6.2)	8.4 ± 1.0	5.7 (2.8;10.4)	0.032
HOMA-IR	1.2 ± 0.2	0.8 (0.6;1.3)	2.5 ± 0.4	1.4 (0.6;2.7)	0.017
GH (µg/L)	1.2 ± 0.3	0.5 (0.1;1.7)	1.1 ± 0.2	0.4 (0.1;1.1)	0.489
IGF-1 (µg/L)	140.5 ± 7.3	133.0 (104.0;178.0)	83.2 ± 4.2	77.3 (56.0;93.0)	<0.001
C-Peptide (ng/mL)	1.6 ± 0.1	1.4 (1.1;1.7)	2.5 ± 0.2	2.1 (1.4;3.2)	<0.001
Cortisol (µg/dL)	15.1 ± 0.8	15.2 (12.2;18.7)	19.7 ± 2.1	13.9 (9.1;21.0)	0.761
C-Reactive Protein (mg/dL)	0.2 ± 0.1	0.1 (0.02;0.2)	0.9 ± 0.1	0.7 (0.4;1.2)	<0.001

TSHRI, TSH index; TSH, thyroid stimulating hormone; TSHRI, TSH index; TT4RI, Thyrotroph T4 Resistance Index.

**Table 3:** Anthropometric, Biochemical, and Hormonal data (Mean ± SE, median, interquartile ranges) in obese patient before and 12 months after bariatric surgery.

	Obese Patients Before Surgery (*n* = 106)		Obese Patients 12 Months After Surgery (*n* = 106)		
	Mean ± SE	Median (IR)	Mean ± SE	Median (IR)	*p*
Weight (Kg)	133.6 ± 25.8	126 (113.2;151.6)	88.0 ± 19.0	84.3 (73.9;100.4)	<0.001
BMI (Kg/m^2^)	49.8 ± 8.9	47.9 (43.6;53.3)	32.7 ± 6.9	31.2 (27.6;36.9)	<0.001
Body fat (%)	49.7 ± 6.0	50.7 (46.8;54.0)	33.8 ± 10.1	34.1 (27.9;36.9)	<0.001
TSH (µU/mL)	3.5 ± 0.3	2.7 (1,6;4.3)	2.2 ± 0.2	2.1 (1.3;2.8)	<0.001
Free T4 (ng/dL)	1.5 ± 0.1	1.4 (1.3;1.6)	1.1 ± 0.1	1.1 (1.0;1.2)	<0.001
TSHRI	3.5 ± 0.1	3.6 (2.9;4.3)	2.5 ± 0.1	2.6 (2.2;2.9)	<0.001
FT4RI	66.0 ± 5.5	50.2 (33.0;81.4)	30.9 ± 2.3	28.6 (19.3;39.2)	<0.001
Fasting Glucose (mg/dL)	102.4 ± 2.8	97.0 (83.0;117.0)	91.6 ± 2.2	86.5 (78.0;94.0)	<0.001
HbA1c (%)	7.2 ± 1.5	5.6 (5.2;6.0)	5.6 ± 0.1	5.3 (5.1;6.0)	<0.001
Fasting Insulin (µIU/mL)	8.4 ± 1.0	5.7 (2.8;10.4)	6.8 ± 0.6	5.4 (3.6;9.5)	0.888
HOMA-IR	2.5 ± 0.4	1.4 (0.6;2.7)	1.5 ± 0.1	1.2 (0.7;2.2)	0.747
GH (µg/L)	1.1 ± 0.2	0.4 (0.1;1.1)	3.3 ± 0.5	2.3 (0.3;5.2)	<0.001
IGF-1 (µg/L)	83.2 ± 4.2	77.3 (56.0;93.0)	112.5 ± 4.2	111.0 (88.0;128.0)	<0.001
C-Peptide (ng/mL)	2.5 ± 0.2	2.1 (1.4;3.2)	2.0 ± 0.1	1.9 (1.5;2.4)	0.921
Cortisol (µg/dL)	19.7 ± 2.1	13.9 (9,1;21,0)	16.0 ± 0.8	15.8 (12.0;18.4)	0.747
C-reactive protein (mg/dL)	0.9 ± 0.1	0.7 (0.4;1.2)	0.1 ± 0.03	0.1 (0.01;0.2)	<0.001

TSHRI, TSH Index; TT4RI, Thyrotroph T4 Resistance Index.

**Table 4:** Generalized estimating equation model examining change of Thyrotroph T4 resistance index (TT4RI) and TSH resistance index (TSHRI) values after bariatric surgery, adjusting for excessive BMI loss in percentage (EBMIL) and C-reactive protein.

	TT4RI			TSHRI		
	B	SE	*p*	B	SE	*p*
Intercept	47.237	5.427	<0.001	3.153	0.160	<0.001
Linear time (months after surgery)	0.113	0.546	0.835	0.004	0.016	0.807
EBMIL	−0.325	0.087	<0.001	−0.011	0.003	<0.001
C-reactive protein (mg/dL)	14.716	5.232	0.005	0.267	0.104	0.011

## Abbreviations

BMI	body mass index
BS	bariatric surgery
CRP	C-reactive protein
CV	coefficient of variation
DL	dyslipidemia
EBMIL	excessive BMI loss in percentage
FT4	free thyroxine
GEE	generalized estimating equations
HTA	hypertension
RYGB	Roux-en-Y gastric bypass
SG	sleeve gastrectomy
TH	thyroid hormone
TSH	thyroid stimulating hormone
TSHRI	TSH index
T4	thyroxine
TT4RI	thyrotroph thyroxine resistance index

## References

[1] Afshin A., Forouzanfar M.H., Reitsma M.B., Sur P., Estep K., Lee A., Marczak L., Mokdad A.H., Moradi-Lakeh M., GBD 2015 Obesity Collaborators (2017). Health Effects of Overweight and Obesity in 195 Countries over 25 Years. N. Engl. J. Med..

[2] Flegal K.M., Kruszon-Moran D., Carroll M.D., Fryar C.D., Ogden C.L. (2016). Trends in Obesity Among Adults in the United States, 2005 to 2014. JAMA.

[3] Ward Z.J., Bleich S.N., Cradock A.L., Barrett J.L., Giles C.M., Flax C., Long M.W., Gortmaker S.L. (2019). Projected U.S. State-Level Prevalence of Adult Obesity and Severe Obesity. N. Engl. J. Med..

[4] Gutierrez-Fisac J.L., Guallar-Castillon P., Leon-Munoz L.M., Graciani A., Banegas J.R., Rodriguez-Artalejo F. (2012). Prevalence of general and abdominal obesity in the adult population of Spain, 2008–2010: The ENRICA study. Obes. Rev..

[5] Ng M., Fleming T., Robinson M., Thomson B., Graetz N., Margono C., Mullany E.C., Biryukov S., Abbafati C., Abera S.F. (2014). Global, regional, and national prevalence of overweight and obesity in children and adults during 1980–2013: A systematic analysis for the Global Burden of Disease Study 2013. Lancet.

[6] Magkos F., Fraterrigo G., Yoshino J., Luecking C., Kirbach K., Kelly S.C., de Las Fuentes L., He S., Okunade A.L., Patterson B.W. (2016). Effects of Moderate and Subsequent Progressive Weight Loss on Metabolic Function and Adipose Tissue Biology in Humans with Obesity. Cell Metab..

[7] Reges O., Greenland P., Dicker D., Leibowitz M., Hoshen M., Gofer I., Rasmussen-Torvik L.J., Balicer R.D. (2018). Association of Bariatric Surgery Using Laparoscopic Banding, Roux-en-Y Gastric Bypass, or Laparoscopic Sleeve Gastrectomy vs Usual Care Obesity Management With All-Cause Mortality. JAMA.

[8] Pena-Bello L., Seoane-Pillado T., Sangiao-Alvarellos S., Outeirino-Blanco E., Varela-Rodriguez B., Juiz-Valina P., Cordido M., Cordido F. (2017). Oral glucose-stimulated growth hormone (GH) test in adult GH deficiency patients and controls: Potential utility of a novel test. Eur. J. Intern. Med..

[9] Pena-Bello L., Pertega-Diaz S., Outeirino-Blanco E., Garcia-Buela J., Tovar S., Sangiao-Alvarellos S., Dieguez C., Cordido F. (2015). Effect of oral glucose administration on rebound growth hormone release in normal and obese women: The role of adiposity, insulin sensitivity and ghrelin. PLoS ONE.

[10] Alvarez-Castro P., Sangiao-Alvarellos S., Brandon-Sanda I., Cordido F. (2011). Endocrine function in obesity. Endocrinol. Nutr..

[11] Biondi B. (2010). Thyroid and obesity: An intriguing relationship. J. Clin. Endocrinol. Metab..

[12] Reinehr T. (2010). Obesity and thyroid function. Mol. Cell. Endocrinol..

[13] Rotondi M., Leporati P., La Manna A., Pirali B., Mondello T., Fonte R., Magri F., Chiovato L. (2009). Raised serum TSH levels in patients with morbid obesity: Is it enough to diagnose subclinical hypothyroidism?. Eur. J. Endocrinol..

[14] Reinehr T., Andler W. (2002). Thyroid hormones before and after weight loss in obesity. Arch. Dis. Child..

[15] Guan B., Chen Y., Yang J., Yang W., Wang C. (2017). Effect of Bariatric Surgery on Thyroid Function in Obese Patients: A Systematic Review and Meta-Analysis. Obes. Surg..

[16] Liu G., Liang L., Bray G.A., Qi L., Hu F.B., Rood J., Sacks F.M., Sun Q. (2017). Thyroid hormones and changes in body weight and metabolic parameters in response to weight loss diets: The POUNDS LOST trial. Int. J. Obes..

[17] Neves J.S., Castro Oliveira S., Souteiro P., Pedro J., Magalhaes D., Guerreiro V., Bettencourt-Silva R., Costa M.M., Cristina Santos A., Queiros J. (2018). Effect of Weight Loss after Bariatric Surgery on Thyroid-Stimulating Hormone Levels in Patients with Morbid Obesity and Normal Thyroid Function. Obes. Surg..

[18] Dall’Asta C., Paganelli M., Morabito A., Vedani P., Barbieri M., Paolisso G., Folli F., Pontiroli A.E. (2010). Weight loss through gastric banding: Effects on TSH and thyroid hormones in obese subjects with normal thyroid function. Obesity.

[19] Juiz-Valina P., Outeirino-Blanco E., Pertega S., Varela-Rodriguez B.M., Garcia-Brao M.J., Mena E., Pena-Bello L., Cordido M., Sangiao-Alvarellos S., Cordido F. (2019). Effect of Weight Loss after Bariatric Surgery on Thyroid-Stimulating Hormone Levels in Euthyroid Patients with Morbid Obesity. Nutrients.

[20] Mullur R., Liu Y.Y., Brent G.A. (2014). Thyroid hormone regulation of metabolism. Physiol. Rev..

[21] Dumitrescu A.M., Refetoff S. (2013). The syndromes of reduced sensitivity to thyroid hormone. Biochim. Biophys. Acta.

[22] Ortiga-Carvalho T.M., Sidhaye A.R., Wondisford F.E. (2014). Thyroid hormone receptors and resistance to thyroid hormone disorders. Nat. Rev. Endocrinol..

[23] Yagi H., Pohlenz J., Hayashi Y., Sakurai A., Refetoff S. (1997). Resistance to thyroid hormone caused by two mutant thyroid hormone receptors beta, R243Q and R243W, with marked impairment of function that cannot be explained by altered in vitro 3,5,3′-triiodothyroinine binding affinity. J. Clin. Endocrinol. Metab..

[24] Jostel A., Ryder W.D., Shalet S.M. (2009). The use of thyroid function tests in the diagnosis of hypopituitarism: Definition and evaluation of the TSH Index. Clin. Endocrinol..

[25] Laurberg P., Knudsen N., Andersen S., Carle A., Pedersen I.B., Karmisholt J. (2012). Thyroid function and obesity. Eur. Thyroid J..

[26] Tjorve E., Tjorve K.M., Olsen J.O., Senum R., Oftebro H. (2007). On commonness and rarity of thyroid hormone resistance: A discussion based on mechanisms of reduced sensitivity in peripheral tissues. Med. Hypotheses.

[27] Laclaustra M., Moreno-Franco B., Lou-Bonafonte J.M., Mateo-Gallego R., Casasnovas J.A., Guallar-Castillon P., Cenarro A., Civeira F. (2019). Impaired Sensitivity to Thyroid Hormones Is Associated With Diabetes and Metabolic Syndrome. Diabetes Care.

[28] Bakdash J.Z., Marusich L.R. (2017). Repeated Measures Correlation. Front. Psychol..

[29] Licenziati M.R., Valerio G., Vetrani I., De Maria G., Liotta F., Radetti G. (2019). Altered Thyroid Function and Structure in Children and Adolescents Who Are Overweight and Obese: Reversal After Weight Loss. J. Clin. Endocrinol. Metab..

[30] Gokosmanoglu F., Aksoy E., Onmez A., Ergenc H., Topkaya S. (2019). Thyroid Homeostasis After Bariatric Surgery in Obese Cases. Obes. Surg..

[31] Bianco A.C., Dumitrescu A., Gereben B., Ribeiro M.O., Fonseca T.L., Fernandes G.W., Bocco B. (2019). Paradigms of Dynamic Control of Thyroid Hormone Signaling. Endocr. Rev..

[32] Castillo M., Freitas B.C., Rosene M.L., Drigo R.A., Grozovsky R., Maciel R.M., Patti M.E., Ribeiro M.O., Bianco A.C. (2010). Impaired metabolic effects of a thyroid hormone receptor beta-selective agonist in a mouse model of diet-induced obesity. Thyroid.

[33] Nannipieri M., Cecchetti F., Anselmino M., Camastra S., Niccolini P., Lamacchia M., Rossi M., Iervasi G., Ferrannini E. (2009). Expression of thyrotropin and thyroid hormone receptors in adipose tissue of patients with morbid obesity and/or type 2 diabetes: Effects of weight loss. Int. J. Obes..

[34] Krause C., Grohs M., El Gammal A., Wolter S., Lehnert H., Mann O., Mittag J., Kirchner H. (2018). Reduced expression of thyroid hormone receptor beta in human nonalcoholic steatohepatitis. Endocr. Connect..

[35] Anselmo J.D., Scherberg N., Dumitrescu A.M., Refetoff S. (2019). Reduced Sensitivity to Thyroid Hormone as a Transgenerational Epigenetic Marker Transmitted Along Human Male Line. Thyroid.

[36] Reinehr T., de Sousa G., Andler W. (2006). Hyperthyrotropinemia in obese children is reversible after weight loss and is not related to lipids. J. Clin. Endocrinol. Metab..

[37] Crujeiras A.B., Goyenechea E., Abete I., Lage M., Carreira M.C., Martinez J.A., Casanueva F.F. (2010). Weight regain after a diet-induced loss is predicted by higher baseline leptin and lower ghrelin plasma levels. J. Clin. Endocrinol. Metab..

[38] Kushchayeva Y.S., Kushchayev S.V., Startzell M., Cochran E., Auh S., Dai Y., Lightbourne M., Skarulis M., Brown R.J. (2019). Thyroid Abnormalities in Patients With Extreme Insulin Resistance Syndromes. J. Clin. Endocrinol. Metab..

[39] Wysocki M., Waledziak M., Hady H.R., Czerniawski M., Proczko-Stepaniak M., Szymanski M., Dowgiallo-Wnukiewicz N., Kozera P., Szeliga J., Orlowski M. (2019). Type 2 Diabetes Mellitus and Preoperative HbA1c Level Have no Consequence on Outcomes after Laparoscopic Sleeve Gastrectomy-a Cohort Study. Obes. Surg..

[40] Glad C.A.M., Svensson P.A., Nystrom F.H., Jacobson P., Carlsson L.M.S., Johannsson G., Andersson-Assarsson J.C. (2019). Expression of GHR and Downstream Signaling Genes in Human Adipose Tissue-Relation to Obesity and Weight Change. J. Clin. Endocrinol. Metab..

[41] Yamauchi I., Sakane Y., Yamashita T., Hirota K., Ueda Y., Kanai Y., Yamashita Y., Kondo E., Fujii T., Taura D. (2018). Effects of growth hormone on thyroid function are mediated by type 2 iodothyronine deiodinase in humans. Endocrine.

[42] Fleseriu M., Hashim I.A., Karavitaki N., Melmed S., Murad M.H., Salvatori R., Samuels M.H. (2016). Hormonal Replacement in Hypopituitarism in Adults: An Endocrine Society Clinical Practice Guideline. J. Clin. Endocrinol. Metab..

[43] Porretti S., Giavoli C., Ronchi C., Lombardi G., Zaccaria M., Valle D., Arosio M., Beck-Peccoz P. (2002). Recombinant human GH replacement therapy and thyroid function in a large group of adult GH-deficient patients: When does L-T(4) therapy become mandatory?. J. Clin. Endocrinol. Metab..

[44] Zhu C., Gao J., Mei F., Lu L., Zhou D., Qu S. (2019). Reduction in Thyroid-Stimulating Hormone Correlated with Improved Inflammation Markers in Chinese Patients with Morbid Obesity Undergoing Laparoscopic Sleeve Gastrectomy. Obes. Surg..

[45] Neves J.S., Souteiro P., Oliveira S.C., Pedro J., Magalhaes D., Guerreiro V., Costa M.M., Bettencourt-Silva R., Santos A.C., Queiros J. (2019). Preoperative thyroid function and weight loss after bariatric surgery. Int. J. Obes..

[46] Biondi B., Kahaly G.J., Robertson R.P. (2019). Thyroid Dysfunction and Diabetes Mellitus: Two Closely Associated Disorders. Endocr. Rev..

